# Conditioned Medium from Human Mesenchymal Stromal Cells: Towards the Clinical Translation

**DOI:** 10.3390/ijms20071656

**Published:** 2019-04-03

**Authors:** Georgy Sagaradze, Olga Grigorieva, Peter Nimiritsky, Nataliya Basalova, Natalia Kalinina, Zhanna Akopyan, Anastasia Efimenko

**Affiliations:** 1 Institute for Regenerative Medicine, Medical Research and Education Center, Lomonosov Moscow State University, 27-10, Lomonosovsky av., Moscow 119191, Russia; go.grigorievaolga@gmail.com (O.G.); nimiritsky@gmail.com (P.N.); natalia_ba@mail.ru (N.B.); zhanna.fbm@gmail.com (Z.A.); efimenkoan@gmail.com (A.E.); 2 Faculty of Medicine, Lomonosov Moscow State University, 27-1, Lomonosovsky av., Moscow 119192, Russia; n_i_kalinina@mail.ru (N.K.)

**Keywords:** regenerative medicine, mesenchymal stem/stromal cells, conditioned medium, secretome, growth factors, manufacturing, quality control, regression analysis

## Abstract

Mesenchymal stem/stromal cells (MSC) remain a promising tool for regenerative medicine as the efficacy of MSC-based cell therapy has been demonstrated for a broad spectrum of indications. Their therapeutic potency is mainly associated with their ability to secrete multiple factors critical for tissue regeneration. Due to comparable effects along with superior safety MSC conditioned medium (MSC-CM) containing a complex of MSC-secreted products is considered a reasonable alternative to cell therapy. However, the lack of standards regulating bioprocessing, use of proper auxiliary materials, and quality control complicates the development of MSC secretome-based therapeutics. In this study, we suggested several approaches addressing these issues. We manufactured 36 MSC-CM samples based on different xeno-free serum-free chemically defined media (DMEM-LG or MSC NutriStem^®^ XF) using original protocols and considered total concentrations of regeneration-associated paracrine factors secreted by human adipose-derived MSC at each time-point of conditioning. Using regression analysis, we retrospectively predicted associations between concentrations of several components of MSC-CM and its biological activity to stimulate human dermal fibroblast and endothelial cell migration in vitro as routine examples of potency assays for cell-based products. We also demonstrated that the cell culture medium might affect MSC-CM biological activity to varying degrees depending on the potency assay type. Furthermore, we showed that regression analysis might help to overcome donor variability. The suggested approaches might be successfully applied for other cell types if their secretome was shown to be promising for application in regenerative medicine.

## 1. Introduction

The application of mesenchymal stem/stromal cells (MSC) in regenerative medicine has been intensively studied in hundreds of clinical trials as these cells represent a promising source of multipotent adult stem and progenitor cells for cell therapy and tissue engineering [1,2]. However, excessive MSC heterogeneity hampers profound cell characterization [3,4,5,6,7]. Several safety concerns related to MSC transplantation still remain because of the potential risk of immune reactions and cancer development [8]. Furthermore, poor engraftment and insufficient viability of transplanted cells restrict their therapeutic efficacy [9,10,11,12].

Therapeutic effects of MSC are generally mediated by various secreted cytokines, growth factors, extracellular matrix proteins and factors involved in matrix remodeling as well as different types of extracellular vesicles [13,14,15,16,17]. MSC-CM that contains cell-secreted products has demonstrated therapeutic benefit for the treatment of ischemic diseases such as myocardial infarction, stroke and acute and chronic hindlimb ischemia, neurodegenerative diseases, spinal cord injury, alopecia, acute and chronic wounds, acute liver injury/failure, lung injury, periodontal tissues injury, male infertility, soft tissue and bone defects [18,19,20,21,22,23]. There are several clinical trials including the use of MSC-CM for hair follicle regeneration [24], fractional carbon dioxide resurfacing wound healing [25] as well as for inflammatory arthritis [26], and multiple sclerosis [27]. MSC-secreted extracellular vesicles that carry regulatory noncoding RNAs were also used as therapeutic agents to stimulate tissue regeneration [28,29,30,31,32]. Thus, MSC secretome is suggested as a novel cell-free medicinal product that can recapitulate the beneficial effects of MSC and has various advantages in overcoming the limitations and risks associated with cell-based therapy [33,34].

However, significant variability of approaches to MSC-CM bioprocessing has a serious impact on experimental outcomes [34,35]. Particularly, the need for disease-specific identity and potency testing due to undefined mechanisms of action of MSC secretome makes development of this class of biopharmaceuticals more complicated, expensive and precarious [1,34,36]. Additionally, the composition of MSC-CM is significantly influenced by donor variability and tissue of MSC origin [37,38] and it should be considered during MSC-CM bioprocessing. In this study, we analyzed how several manufacturing features such as duration of cell conditioning or selection of particular growth medium might influence the composition of human adipose-derived MSC-CM as well as its biological activity in several potency assays routinely used for the development of cell-based therapy products. We then performed regression analysis to estimate power to predict biological activity of MSC-CM samples despite high donor variability and complex composition of MSC-CM.

## 2. Results

### 2.1. Characterization of Mesenchymal STEM/Stromal Cells

Culturing of cells harvested from subcutaneous adipose tissue in the medium supporting the growth of undifferentiated MSC allowed us to get a population of fibroblast-like cells with characteristics of MSC according to criteria from the International Society for Cellular Therapy Statement at the 2nd to 3rd passages [39,40,41]. MSC expressed CD73, CD90, CD 105, and did not express CD14, CD20, CD34, and CD45 (Figure S1). The isolated cells demonstrated bone mineralization and neutral lipid accumulation in the appropriate culture medium and conditions, thus confirming the ability to differentiate into osteogenic and adipogenic lineages (Figure S2).

### 2.2. Development of MSC-CM Bioprocessing Protocol

Two growth media were selected for producing MSC-CM. First, chemically defined low glucose DMEM (DMEM-LG) was chosen as a conventionally used medium for MSC culture. Second, among commercially available media designed to specifically support growth of undifferentiated MSC we selected a defined, xeno-free, serum-free MSC NutriStem medium (Biological Industries, Israel). It is important to note that only basal media without nutrimental supplements (FBS or NutriStem supplement) were used for MSC conditioning.

To develop the original MSC-CM bioprocessing protocol, we firstly assessed cell viability dynamics during long-term conditioning. Both media supported appropriate viability of MSC during long-term conditioning in supplement-free conditions (all cultures contained ≥ 70% of viable cells at least for 12 days) (Table 1). The level of cell proliferation in these culture conditions was relatively low due to the lack of supplements and did not differ between the media.

**Figure 1:**
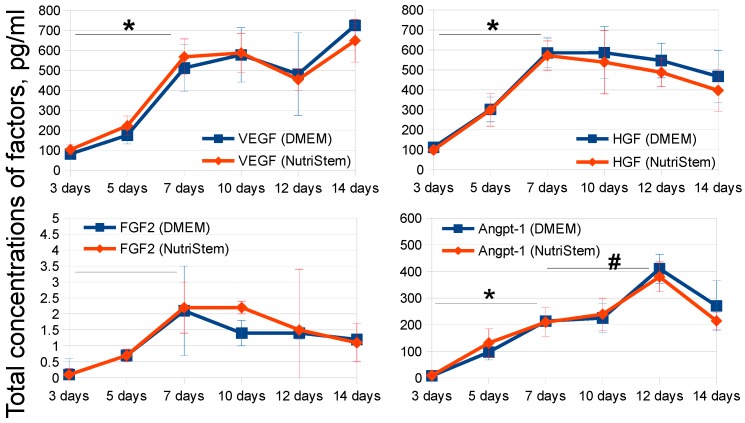
Total growth factor concentrations in DMEM- (blue lines) and NutriStem-based (red lines) MSC-CM samples (*n* = 3) collected independently at each timepoint of MSC conditioning. The data are presented as mean concentrations in pg/mL ± SD. MSC-CM samples were analyzed in three independent replicates for both media. Only MSC-CM samples based on the same medium were compared. * *p* < 0.05 compared to the factor concentration at day 3, # *p* < 0.05 compared to the factor concentration at day 7.

To determine the appropriate period of MSC conditioning, we analyzed total concentrations of selected growth factors in MSC-CM on certain days. According to the literature data and our previous results [42,43,44], we chose four key growth factors, which were shown to substantially contribute to positive effects of MSC on reparative and regenerative processes in damaged tissues, namely vascular endothelial growth factor (VEGF), hepatocyte growth factor (HGF), fibroblast growth factor 2 (FGF2), and angiopoetin-1 (Angpt-1). Peak factor concentrations were mostly reached at days 7 or 10 in both media (Figure 1). Due to minor differences between the concentrations of growth factors at days 7 and 10 and the substantial reduction of MSC-CM manufacturing duration, the 7-day protocol of MSC conditioning was selected for further experiments.

**Figure 2:**
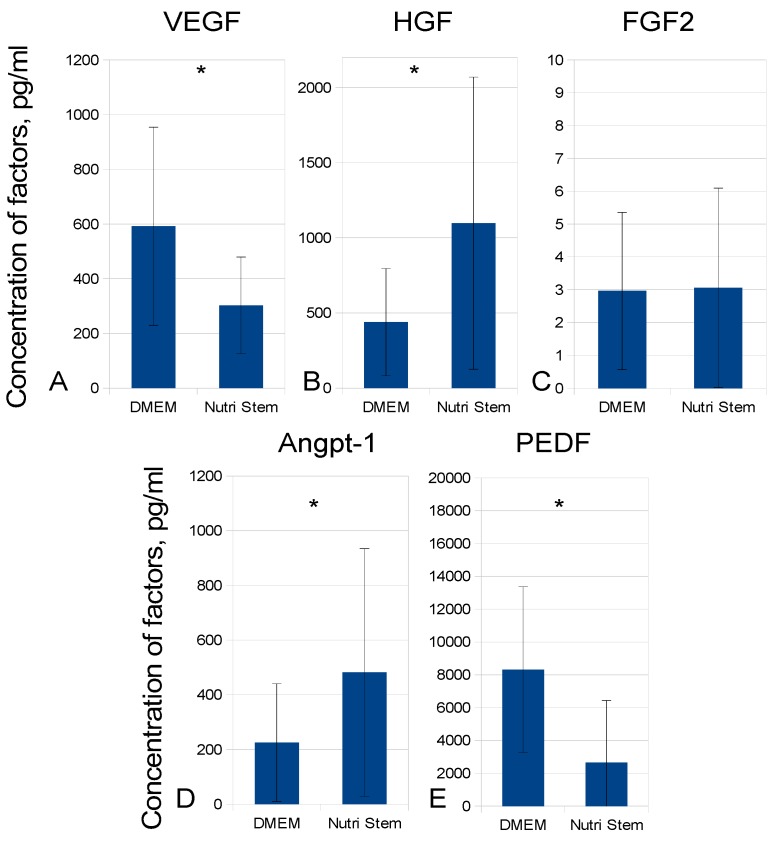
Comparison of growth factor concentrations in MSC-CM based on different MSC culture media at day 7. The data are presented as mean concentrations in pg/mL ± SD. * *p* < 0.05 after Student’s *t*-test analysis. All manufactured 36 MSC-CM samples were analyzed.

To establish whether MSC growth medium may have influenced the composition of MSC-CM, we compared factor concentrations in MSC-CM samples (*n* = 36) manufactured using DMEM-LG or NutriStem. Pigment-epithelial derived factor (PEDF) was also included in the analysis as overrepresented MSC-CM protein possibly counterbalancing its angiogenic effects. The concentration of VEGF and PEDF were higher in DMEM-LG conditioned medium while HGF and Angpt-1 concentrations were higher in NutriStem MSC-CM samples (Figure 2). Thus, a possible impact of the cell culture medium on MSC-CM composition was considered in further experiments. It is worth noting that the means of growth factor concentrations in the 36 analyzed samples differed from the values presented in Figure 1, which obviously indicates the reason for analyzing large sample groups.

### 2.3. Evaluation of MSC-CM Functional Activity in Vitro

**Figure 3:**
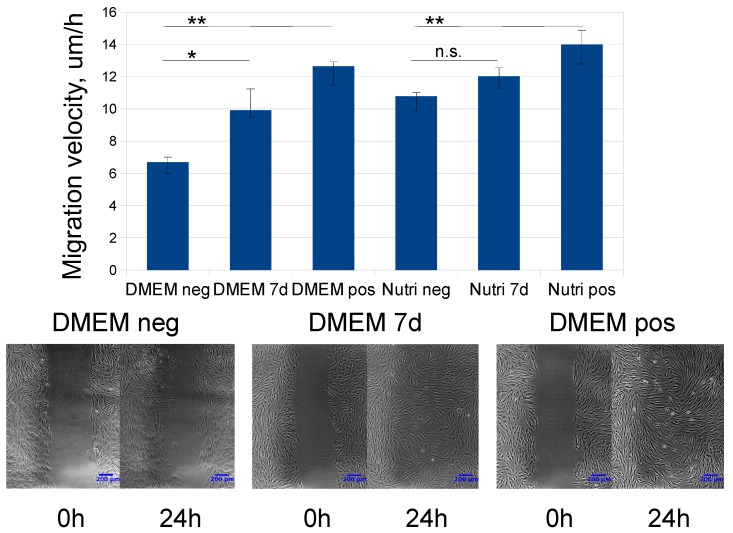
Human dermal fibroblast migration (scratch wound assay) stimulated by MSC-CM obtained after 7 days of long conditioning in DMEM-LG or NutriStem. Graph: DMEM neg—basal medium DMEM-LG (*n* = 5), DMEM pos—DMEM-LG + 10% FBS (*n* = 5), Nutri neg—basal NutriStem medium (*n* = 5), Nutri pos—NutriStem + 10% NutriStem Supplement (*n* = 5), DMEM 7d (*n* = 12) and Nutri 7d (*n* = 15)—MSC-CM samples obtained after conditioning of MSC for 7 days. After the scratch was made, either samples or controls were added to the cells for 24 h. Data are presented as medians and 25th-, 75th percentiles of human dermal fibroblast migration in um/h. * *p* < 0,05; ** *p* < 0.01; n.s.—*p* = 0.08. Low panel: Scratch wound closure at the start point (0 h) and after 24 h; scalebar marks 200 um.

**Figure 4:**
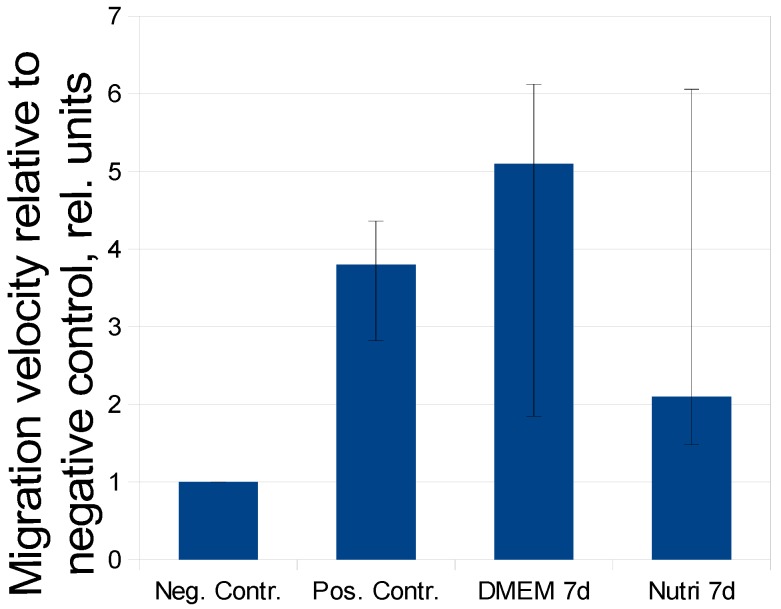
Directional migration of human endothelial cells EA.hy926 over a period of 4 h toward MSC-CM samples obtained after 7 days of long conditioning. Neg. Contr—basal medium DMEM-LG (*n* = 3), Pos. Contr—DMEM-LG + 10% FBS (*n* = 3), DMEM 7d (*n* = 6) and Nutri 7d (*n* = 4)—MSC-CM samples. Basal DMEM-LG was used as a negative control for all the CM samples due to a lower relative potency of NutriStem supplemented medium compared to basal medium (1.59 fold; data are not presented). The relative cell migration velocity is presented. The data is displayed as medians and 25th, 75th percentiles. * *p* < 0.05.

To assess functional activity of MSC-CM samples, we used two well-established in vitro models reflecting important processes for tissue repair. Firstly, non-directional migration of human dermal fibroblasts stimulated by MSC secreted products were analyzed in the scratch assay. Secondly, directional migration of human endothelial cells stimulated by MSC-CM was measured using the automated xCELLigence system to allow real-time registration of the process. Both the studied MSC-CM types stimulated human dermal fibroblast migration as well as human endothelial cell migration, however, the effects of NutriStem MSC-CM samples were less expressed (Figure 3 and Figure 4).

### 2.4. Development of the Prediction Model for MSC-CM Potency Using Regression Analysis

**Figure 5:**
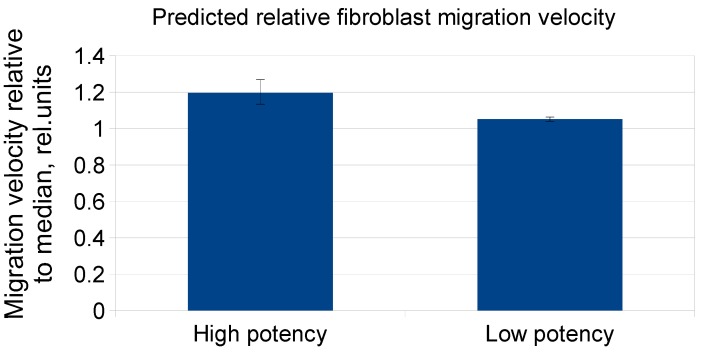
Comparison of two groups of MSC-CM samples that differed in potency. The data are presented as medians, 25th, and 75th percentiles, *p* < 0.086 between these groups, *n* = 10 for High potency group, *n* = 2 for Low potency group.

To define the factors associated with the potency of MSC-CM samples in the studied in vitro models, we performed regression analysis. It is important to note that the type of MSC growth medium was also considered as a possible predictor of the potency due to its discrepant influence on factor concentrations. Surprisingly, we showed that Angpt-1 concentration was the most potent predictor of human dermal fibroblast migration stimulated by MSC-CM. MSC growth medium was not considered a reliable predictor (*p* = 0.448). Using only Angpt-1 concentrations we divided MSC-CM sample into groups with differing potency (*p* < 0.086) (Table 2, Figure 5).

It was found that neither analyzed factors were untrustworthy to predict the potency of MSC-CM to stimulate endothelial cell migration (Table 3). Therefore, considering possible associations of more than one factor with MSC-CM effects and the ambiguous influence of the cell growth medium on their concentrations, we performed regression analysis for DMEM-LG and NutriStem-based MSC-CM samples separately.

**Figure 6:**
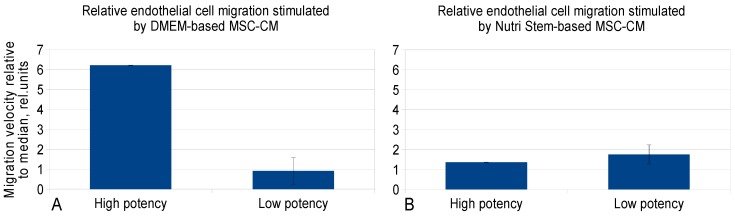
Comparison of two groups of MSC-CM samples that differed in potency to stimulate human endothelial cell migration. **A**—Samples manufactured using DMEM-LG, *n* = 1 for High potency group, *n* = 4 for Low potency group; **B**—NutriStem, *n* = 1 for High potency group, *n* = 4 for Low potency group. The data are presented as means ± SD.

It was demonstrated that FGF2 concentration analysis was sufficient to classify DMEM-LG MSC-CM samples on the basis of their potency to stimulate the migration of human endothelial cells (Table 4, Figure 6A). However, the potency predictors of NutriStem MSC-CM were not discovered (Table 5, Figure 6B).

Taken together, the use of regression analysis might allow for the prediction of the potency of MSC-CM samples using only concentrations of a restricted number of secreted factors. This could simplify MSC-CM quality control as well as the donor selection procedure.

However, during analysis one should take into account possible interactions between distinct growth factors. In this study, several interactions were considered based on discovered significant rank correlations between HGF release and FGF2, VEGF, PEDF concentrations. Nevertheless, their inclusion in the model did not change the results of the analysis seriously.

## 3. Discussion

According to the current concepts, MSC could orchestrate tissue development, maintenance and repair, mostly by producing multiple secretory factors [45,46,47,48,49,50,51]. Therefore, the application of MSC-CM might be an effective strategy for regenerative medicine. To date, several MSC-derived conditioned media were tested on various diseases and many of them showed positive results [22]. An additional benefit is that MSC-CM might be an off-the-shelf material that could be used to treat patients promptly without MSC isolation and subsequent culture. However, despite the clear benefits of using MSC-CM for regenerative medicine, several issues must be addressed before its successful clinical application. Among them, one of the most important is a lack of common recommendations or standards for bioprocessing and quality control of MSC secretome-based therapeutics [52]. In this study, we suggested universal approaches that might help to overcome these issues and, finally, optimize the development of MSC-CM based products in general.

To develop the MSC-CM bioprocessing protocol we selected two growth media for MSC conditioning: DMEM due to its wide application in the manufacturing of MSC-based cell products, including the clinical trials [53] and NutriStem as one of the specific media with great potential to support growth and functional properties of undifferentiated human MSC. Both media were chemically defined, available as GMP-grade media and appropriate for cell manufacturing. It is critical to note that we used only basic media for MSC conditioning without adding the specific nutrimental supplements, because of their high risk influence on the biosafety of a final product and serious challenges for further clinical translation [54]. We showed that both media supported appropriate viability of MSC during the long-term conditioning in supplement-free conditions.

Due to complex composition of MSC-CM, it was necessary to focus on components that would reflect its regenerative potency for a specific condition. In our study, we selected several factors crucial for MSC secretome-mediated tissue regeneration, which was also confirmed by our previous data [42,43,44]. VEGF, an important pro-angiogenic and neurotrophic factor [43], is produced by MSC and served as one of the main mediators in MSC interaction with endothelial cells. Particularly, VEGF expression by MSC was associated with the stimulation of angiogenesis and endothelial cell proliferation in both small and large animal studies [55], and blocking of VEGF in MSC-CM resulted in significant reduction in its ability to stimulate angiogenesis [42,44]. On the contrary, PEDF, another factor highly represented in MSC secretome [56], has anti-angiogenic effects; the ratio of VEGF/PEDF produced by MSC may indicate the ability of the cells to stimulate angiogenesis [57]. MSC also secretes HGF, a factor with angiogenic, anti-apoptotic and immune modulating activity shown to be critical in several in vitro and in vivo therapeutic effects of MSC [43,58]. FGF2, a strong mitogenic and pro-migratory factor for fibroblasts, has the potential to promote angiogenesis and to increase survival and proliferation of stem cells in vivo [59,60].

Obviously, the panel of factors evaluated in MSC-CM may vary depending on potential therapeutic mechanisms for a specific disease. Particularly, in several injury models, either a complex of growth factors from MSC-CM as well as other bioactive components of MSC secretome (i.e., extracellular vesicles) might confer the principal potency for restoration [61]. The components of MSC-CM also might differ by their accumulation dynamics in cell growth medium, so we considered the peak concentrations of several factors to improve the performance of the bioprocessing protocol. Importantly, it was demonstrated that the type of a cell growth medium affected secretory potential of MSC, which was in accordance with literature data [62,63].

To optimize specific biological activity testing of MSC-CM, we suggested to perform regression analysis. Based on initial results of the testing on biological models, this approach can help to select MSC-CM factors, which concentrations are closely associated with the specific biological activity of MSC-CM. This might make possible to predict specific activity using precise instrumental analysis, thus bypassing expensive and laborious biological assays. Accordingly, the estimation of the concentrations of irrelevant MSC-CM factors might be omitted.

In this study, we demonstrated that FGF2 concentration in MSC-CM was associated with DMEM-based CM-stimulated endothelial cell migration velocity. Importantly, these results were concordant with the mechanisms of action of this factor [60]. However, the similar results were not obtained for NutriStem-based MSC-CM samples possibly due to direct substantial influence of the basal medium on the potency. Unexpectedly, it was also demonstrated that Angpt-1 concentration in MSC-CM was inversely associated with CM-stimulated fibroblast migration velocity independently of the type of cell culture medium. Indeed, it did not fit with the well-established superior pro-migratory potential of FGF2 [59]. Nevertheless, these results might be interpreted as evidence that Angpt-1 concentrations predicted activity to stimulate fibroblast migration for the set composed of DMEM and NutriStem MSC-CM samples, even despite lack of established biological associations. Use of such an approach allows to make MSC-CM bioprocessing protocols more flexible in relation to the choice of raw materials.

Noteworthy, regression analysis used in this study has several limitations. It is impossible to consider all the interactions of soluble factors in MSC-CM. Furthermore, the analyzed components of MSC-CM could have synergistic or antagonist effects on each other which might distort their real impact on biological activity of MSC-CM, but couldn’t be revealed by mathematical modeling. Nevertheless, use of the regression analysis might be rational. We interpreted the release of factors as endpoints that might vary due to donor-associated heterogeneity or influence of other MSC-CM components. The obtained results would reflect associations of factor concentrations with specific biological activity of a sample considering its variability but possibly irrelevant to its biological function.

Taken together, our results have demonstrated the applicability of the approaches to MSC-CM bioprocessing and quality control optimization. Despite hurdles associated with the development of MSC secretome-based products, we tried to circumvent the high variability between donors and indicate a practical way to the choice of relevant quality control criteria. We suggest that these approaches might be adapted for other cell types and their secretomes promising for application in regenerative medicine.

## 4. Materials and Methods

### 4.1. Patients

Thirty patients were included in the study. They underwent surgery because of general surgical pathology, kidney and bladder revision. Exclusion criteria included any patient aged less than 18 years and more than 70 years, autoimmune pathologies, cancer (even in the past history), acute or chronic inflammatory disease, type 2 diabetes mellitus, acute myocardial infarction in the previous six months, long-term hormone or antibiotics therapy, hematological disorders, stroke or craniocerebral injury in the previous 12 months, polyvalent allergy and pregnancy. The clinical features of the patient cohort are presented in Table S1. All procedures performed with tissue samples from patients were in accordance with the Declaration of Helsinki and approved by the Ethics Committee of Lomonosov Moscow State University (IRB00010587), protocol #4 (2018). Each donor participated in the study, signed an informed written consent form for harvesting and using adipose tissue samples as well as for handling clinical data for research purposes.

### 4.2. Isolation of Adipose-Derived MSC

Subcutaneous adipose tissue samples (0.5–5 mL) harvested during surgery were homogenized and digested in collagenase I (200 U/mL, Worthington Biochemical) and dispase (40 U/mL, Sigma, St. Louis, MO, USA) solutions under agitation for 30–40 min at 37 °C. Then tissue was centrifuged at 200 g for 10 min; supernatant was discarded. Erythrocytes were removed from the MSC pellet by a brief hypoosmotic shock. Then, cell suspension was filtered through a sieve (BD Falcon Cell Strainer, 100 um, Franklin Lakes, NJ, USA) and centrifuged at 200 g for 10 min. The final pellet was resuspended in culture medium. The cells were cultured in standard conditions (5% CO^2^; 37 °C) in Advance Stem Cell Basal Medium (ASCBM, HyClone, Marlborough, MA, USA) with 10% of Advance Stem Cell Growth Supplement (HyClone), and 100 U/mL penicillin/streptomycin (HyClone). Unattached cells were washed off 24 h after isolation, and then, medium was changed every 3 to 4 days. The yield of cells was 4–7 × 10^4^ of attached cells per ml of tissue. Cells were passaged at 70% confluency using HyQTase solution (HyClone).

### 4.3. Collection of MSC conditioned medium

MSC at 4–5th passages were seeded with a density of 3 × 10^3^ cells/cm^2^ on uncoated culture plastic (Corning) and were cultured to 70–80% confluence in 100 mm culture dishes. Then MSC were washed thoroughly 3 times using 10 mL of HBSS without Ca2^+^ and Mg2^+^, and replenished with MSC NutriStem XF Basal Medium (Nutristem, Biological Industries, Beit-Haemek, Israel) or DMEM with low glucose (DMEM-LG, HyClone). Cells were cultured in standard conditions (5% CO_2_; 37 °C) for different time periods. Then, conditioned media samples were collected, centrifuged at 3000 rpm for 10 min at 4 °C to remove cell debris, then frozen in aliquots at −70 °C. 36 MSC-CM samples were manufactured (*n* = 21 for DMEM-LG, *n* = 15 for NutriStem).

### 4.4. Analysis of Cell Viability

The quantity and viability of MSC were assessed at the end of the experiment by staining with trypan blue solution. Cell viability was interpreted as the ability of viable cells to eject the trypan blue stain. The amount of viable (bright) and dead (blue) cells was evaluated using the automated cell counter Countess (Invitrogen, Carlsbad, CA, USA). Cell viability was determined as a ratio of viable cells to the total cell amount. To estimate cell viability during long-term conditioning, the cells were grown in separate dishes and were analyzed in certain time points independently.

### 4.5. MSCs Immunophenotyping and Differentiation Assays

To confirm that MSC were multipotent mesenchymal stromal cells we analyzed their immunophenotype according to the published criteria [39]. Cells were stained with anti- CD73, CD90, CD105, CD14, CD20, CD34, CD45 antibodies and appropriate isotype control antibodies (MSC Phenotyping Kit, Miltenyi Biotec, Bergisch Gladbach, Germany) and analyzed using flow cytometry.

The potential of MSC for osteogenic and adipogenic differentiation was tested using standard techniques in vitro. Briefly, osteogenic differentiation was induced by plating 6 × 10^4^ MSC on a 24-well plate and incubated in an Advance Stem Cell Osteogenic Medium (HyClone) containing 10% Advance Stem Cell Supplement and 100 U/mL penicillin/streptomycin for 21 days. Differentiation efficiency was analyzed using Alizarin Red S staining for calcium accumulation. Adipogenic differentiation was induced by the incubation of MSC in Advance Stem Cell Adipogenic Medium (HyClone) containing 10% Advance Stem Cell Supplement and 100 U/mL penicillin/streptomycin for 18 days. Cells accumulated intracellular lipids were analyzed using Oil-Red-O staining.

### 4.6. Analysis of Concentrations of Growth Factors in MSC-Conditioned Medium by ELISA

The concentrations of VEGF, HGF, FGF2, Angpt-1 and PEDF in MSC-CM samples were analyzed using ELISA (R&D Systems) according to the manufacturer’s instructions. Factor concentrations were determined in conditioned medium collected from independent cell culture plates. The cells isolated were seeded on cell culture dishes. Then, the medium was collected, centrifuged and frozen. The total release of factors was calculated based on the analysis of secretome samples at every time point.

### 4.7. Migration of Fibroblasts in the Scratch Assay

Scratch assay is a specific test with an artificial wound scrapped mechanically in a confluent cell monolayer. Fibroblasts activated by an empty plastic area move from the edge to the center of the wound up to wound closure. Several factors like FBS or other factors, contained in MSC-CM, might have an influence on cell motility. Human skin fibroblasts were grown to confluent in 24-well plates in DMEM-LG containing 10% of the FBS. Then, fibroblasts were deprived in DMEM with 0% FBS for 24 h. Cell monolayers were scratched with a 1 mL pipette tip and briefly rinsed. Then, the sample of MSC-CM or DMEM-LG supplemented with 10% FBS as a positive control or serum-free DMEM-LG as a negative control were added. Following this, culture plates were transferred onto the microscopic stage of a motorized Nikon Ti inverted microscope (Nikon, Japan) equipped with the 5x objective, on-stage culture box, temperature controller set to 37 °C and continuous carbogen administration unit. The time-lapse series was continuously acquired every 15 min over 24 h using a cooled CCD camera (Nikon, Tokyo, Japan) and the “Mark and Find” application in NIS Elements (Nikon, Japan) to achieve simultaneous image acquisition in all 24 wells of the plate. This frequency ensured that in each series two successive displacements of a cell were resolved and all cell divisions were captured to be excluded from the analysis later on. The time series were analyzed by manual tracking of all cells on the edge of the experimental wounds and their velocity was measured in two randomly chosen positions of the wounded areas using free ImageJ software, Madison, WI, USA. Routinely, 50 cells were tracked for each data point.

### 4.8. Endothelial Cell Migration Analysis in the xCELLigence RTCA DP System

To analyze endothelial cell migration using the CIM-Plate 16, human endothelial cells EA.hy926 were cultured in DMEM with high glucose supplemented with 10% FBS; cells were deprived in serum-free DMEM for 6 to 8 h prior to the experiment and seeded 30 × 10^4^ cells in 50 uL of serum-free DMEM per well into the upper chambers of the CIM-Plate 16. Samples of MSC-CM (160 uL per well) were placed into the lower chambers. DMEM supplemented with 10% FBS was used as a positive control and serum-free DMEM served as a negative control. Then CIM-Plate 16 was placed in the RTCA DP Instrument (Roche, Basel, Switzerland) equilibrated in a CO_2_ incubator. Endothelial cell migration was continuously monitored using the RTCA DP Instrument. MSC-CM provided a strong chemoattractant signal, which induced the directional migration of endothelial cells through the micropores of the CIM-Plate 16. Migrating cells were detected by the electronic sensing microelectrodes, producing changes in the measured Cell Index values. Time-dependent cell migration was monitored over 4 h. All experiments were performed in duplicates. The RTCA Software 1.2 was used to calculate Cell Index values for MSC-CM-mediated endothelial cell migration.

### 4.9. Regression Analysis

The samples were randomized to training and validation groups prior to analysis (2:1 for fibroblast migration and initial endothelial cell migration, 1.5:1 for cell growth medium-specific endothelial cell migration). Growth factor concentrations in MSC-CM samples were standardized prior to regression model building. We used the Python StatsModels library and the Logit model, to perform regression analysis. For fibroblast scratch assay we categorized our data into two groups according to their specific activity: The first group consisted of samples with below median activity (*n* = 18), the second group included samples with equal to or above median activity (*n* = 18). For endothelial cell migration we split our data into two groups similarly: The first group consisted of 20 samples, the second included 11 more potent samples. We selected factor concentrations as well as the type of cell culture medium as independent variables.

### 4.10. Statistical Analysis

Statistical analysis was performed using RStudio. The normality was tested using the Shapiro–Wilk test. Normally distributed data were compared using a Student’s t-test; data that were not normally distributed were compared using the Mann–Whitney U-test or the Kruskal–Wallis test. Multiple comparisons were made using the Kruskall–Wallis test with subsequent application of Dunn criteria. Correlations were calculated using the Hmisc R package. Statistical significance was defined as *p*-value <0.05.

## Figures and Tables

**Table 1:** Viability of mesenchymal stem/stromal cells during long-term conditioning in two basal media. The data from three independent experiments have been presented as means ± SD.

Time, Days/Growth Medium	3 Days	5 Days	7 Days	10 Days	12 Days	14 Days
DMEM	80.3 ± 1.5	77.0 ± 2.6	78.3 ± 3.2	71.7 ± 3.8	90.7 ± 6.7	59.7 ± 11.7
Nutri Stem	76.0 ± 4.0	77.3 ± 5.0	73.3 ± 11.2	72.0 ± 8.5	73.7 ± 13.1	76.0 ± 5.2

**Table 2:** Prediction model for MSC-CM sample potency to stimulate human dermal fibroblast migration; *n* = 24 for train group, *n* = 12 for validation group.

Predictor.	Coefficient	*P*-Value	95% Conf. Interval
Intercept	0.58	0.53	−1.9; 1.25
VEGF	0.67	0.46	−1.35; 0.99
HGF	0.80	0.27	−0.82; 1.62
FGF2	0.67	0.44	−0.74; 2.53
Angpt-1	−1.83	0.08	−3.81; 0.78
PEDF	1.14	0.15	−0.48; 1.64
Medium type	−1.17	0.45	−2.69; 2.62
Single factor analysis			
Predictor	Coefficient	P-value	95% Conf. Interval
Intercept	0.09	0.85	−0.85; 1.03
Angpt1	−1.16	0.11	−2.59; 0.27

**Table 3:** Prediction model for MSC-CM sample potency to stimulate human endothelial cell migration; *n* = 20 for train group, *n* = 11 for validation group.

Predictor	Coefficient	P-Value	95% Conf. Interval
Intercept	2.75	0.61	−7.7; 13.2
VEGF	2.76	0.31	−2.52; 8.04
HGF	0.66	0.72	−2.96; 4.29
FGF2	8.78	0.69	−34.11; 51.67
Angpt1	−1.46	0.70	−9.01; 6.09
PEDF	0.89	0.54	−1.99; 3.77
Medium type	−4.10	0.70	−24.8; 16.58

**Table 4:** Prediction model for DMEM-LG MSC-CM sample potency to stimulate human endothelial cell migration; *n* = 8 for train group, *n* = 5 for validation group.

Predictor	Coefficient	P-Value	95% Conf. Interval
Intercept	17.70	0.44	−26.83;62.23
VEGF	−1.12	0.78	−8.93;6.69
HGF	−1.18	0.68	−6.81;4.46
FGF2	60.19	0.44	−92.44;212.81
Angpt1	1.21	0.89	−15.59;18
PEDF	0.81	0.66	−2.82;4.44
Single factor analysis			
Predictor	Coefficient	P-value	95% Conf. Interval
Intercept	13.08	0.17	−5.7;31.86
FGF2	48.78	0.14	−15.7;113.27

**Table 5:** Prediction model for NutriStem MSC-CM sample potency to stimulate human endothelial cell migration; *n* = 7 for train group, *n* = 5 for validation group.

Predictor	Coefficient	P-Value	95% Conf. Interval
Intercept	−1.04	0.95	−30.57;28.49
VEGF	4.81	0.76	−25.56;35.17
HGF	−0.72	0.96	−28.07;26.63
FGF2	−1.73	0.91	−32.6;29.13
Angpt1	2.54	0.81	−18.66;23.8
PEDF	0.45	0.98	−37.8;38.7
Single factor analysis			
Predictor	Coefficient	P-value	95% Conf. Interval
Intercept	−0.06	0.99	−10.79;10.67
VEGF	7.68	0.42	−10.84;26.2

## Supplementary Materials

Supplementary materials can be found at https://www.mdpi.com/1422-0067/20/7/1656/s1.

## Abbreviations

MSC	mesenchymal stem/stromal cells
CM	conditioned medium
DMEMLG	Dulbecco’s Modified Eagle Medium with low glucose content
VEGF	vascular endothelial growth factor
HGF	hepatocyte growth factor
FGF2	basic fibroblast growth factor
Angpt-1	angiopoietin-1
PEDF	pigment epithelial cells derived factor
FBS	fetal bovine serum
