# Supplementary material for: Conditioned Medium from Human Mesenchymal Stromal Cells: Towards the Clinical Translation
Source: Int J Mol Sci. 2019 Apr 3;20(7):1656. doi: 10.3390/ijms20071656 (PMC6479925; doi:10.3390/ijms20071656)
Supplement: Supplementary file 1 [file ijms-20-01656-s001.pdf]

Table S1. Clinical characteristics of patients.

**Characteristics**	**Men**	**Women**
Number of patients	30	
	16	14
Age, years	52,8 ±14,6	
	52,2 ±15,9	53,4 ±12,8
Hypertension, n (%)	13 (43,3%)	
Carbohydrate metabolism disorders, n (%)	0 (0%)	
Dyslipidemia, n (%)	0 (0%)	
Obesity, n (%)	0 (0%)	
BMI, kg/m2	23,4 ±2,3	
	23,1 ±1,8	23,7 ±2,7
Smoker, n (%)	5 (16,7%)	

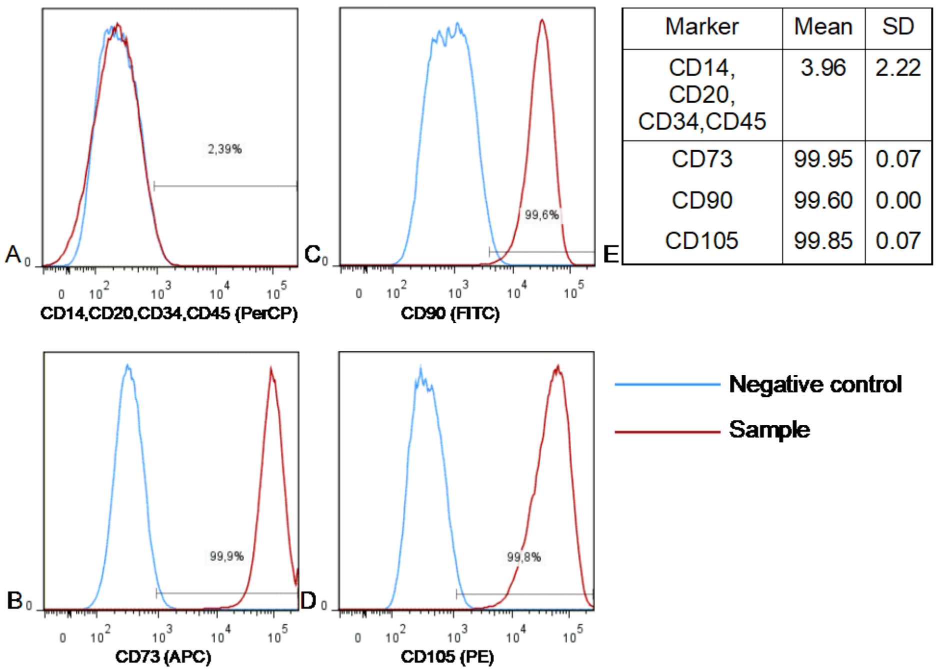

Marker	Mean	SD
CD14, CD20, CD34,CD45	3.96	2.22
CD73	99.95	0.07
CD90	99.60	0.00
CD105	99.85	0.07

Figure S1. Phenotypic characterization of adipose-derived MSC. Flow cytometric analysis was performed as described in “Materials and Methods”. A – analysis of CD14, CD20, CD34, CD45 expression (PerCP); B – CD73 (APC); C – CD90 (FITC); D – CD105 (PE). E – Average percent and SD of cells stained positive for the markers. The results from 2 donors were presented in the table.

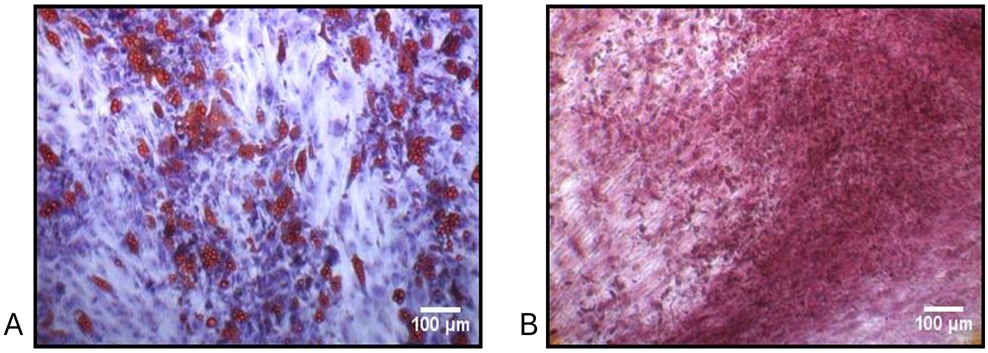

Figure S2. Adipogenic (A) and osteogenic (B) differentiation of human adipose-derived MSC. Scalebars mark 100 um.
